# Inference of a Probabilistic Boolean Network from a Single Observed Temporal Sequence

**DOI:** 10.1155/2007/32454

**Published:** 2007-05-07

**Authors:** Stephen Marshall, Le Yu, Yufei Xiao, Edward R Dougherty

**Affiliations:** 1 Department of Electronic and Electrical Engineering, Faculty of Engineering, University of Strathclyde, Glasgow, G1 1XW, UK; 2 Department of Electrical and Computer Engineering, Texas A&M University, College Station, TX 77843-3128, USA; 3 Computational Biology Division, Translational Genomics Research Institute, Phoenix, AZ 85004, USA; 4 Department of Pathology, University of Texas M. D. Anderson Cancer Center, Houston, TX 77030, USA

## Abstract

The inference of gene regulatory networks is a key issue for genomic signal processing. This paper addresses the inference of probabilistic Boolean networks (PBNs) from observed temporal sequences of network states. Since a PBN is composed of a finite number of Boolean networks, a basic observation is that the characteristics of a single Boolean network without perturbation may be determined by its pairwise transitions. Because the network function is fixed and there are no perturbations, a given state will always be followed by a unique state at the succeeding time point. Thus, a transition counting matrix compiled over a data sequence will be sparse and contain only one entry per line. If the network also has perturbations, with small perturbation probability, then the transition counting matrix would have some insignificant nonzero entries replacing some (or all) of the zeros. If a data sequence is sufficiently long to adequately populate the matrix, then determination of the functions and inputs underlying the model is straightforward. The difficulty comes when the transition counting matrix consists of data derived from more than one Boolean network. We address the PBN inference procedure in several steps: (1) separate the data sequence into "pure" subsequences corresponding to constituent Boolean networks; (2) given a subsequence, infer a Boolean network; and (3) infer the probabilities of perturbation, the probability of there being a switch between constituent Boolean networks, and the selection probabilities governing which network is to be selected given a switch. Capturing the full dynamic behavior of probabilistic Boolean networks, be they binary or multivalued, will require the use of temporal data, and a great deal of it. This should not be surprising given the complexity of the model and the number of parameters, both transitional and static, that must be estimated. In addition to providing an inference algorithm, this paper demonstrates that the data requirement is much smaller if one does not wish to infer the switching, perturbation, and selection probabilities, and that constituent-network connectivity can be discovered with decent accuracy for relatively small time-course sequences.

## 

[1,2,3,4,5,6,7,8,9,10,11,12,13,14,15,16,17,18,19,20,21,22,23,24,25,26,27,28,29,30,31]
